# Chemical Composition and Biological Activities of *Psilocybe* Mushrooms: Gaps and Perspectives

**DOI:** 10.3390/ph18070989

**Published:** 2025-07-01

**Authors:** Mateus A. Luz, Hellen V. S. Guedes, Antônio B. M. Bisneto, Raquel A. de Jesus, Taynah P. Galdino, Lucas C. Oliveira, Victor Ignacio Afonso, Marcus Vinícius L. Fook, Antônio G. B. Lima, Suedina M. de L. Silva, Maria C. M. Torres

**Affiliations:** 1 Department of Chemistry, Paraíba State University, Campina Grande 58429-500, Paraíba, Brazil; mateus.luz@aluno.uepb.edu.br (M.A.L.); hellen.guedes@aluno.uepb.edu.br (H.V.S.G.); 2 Northeast Biomaterials Evaluation and Development Laboratory, CERTBIO, Academic Unit of Materials Engineering, Federal University of Campina Grande, Av. Aprígio Veloso, 882-Bodocongó, Campina Grande 58429-900, Paraíba, Brazil; antonio.bisneto@aluno.uepb.edu.br (A.B.M.B.); raquel.albino@estudante.ufcg.edu.br (R.A.d.J.); taynah.galdino@certbio.ufcg.edu.br (T.P.G.); lucas.cordeiro@certbio.ufcg.edu.br (L.C.O.); marcus.liafook@certbio.ufcg.edu.br (M.V.L.F.); 3 Department of Physiotherapy, Paraíba State University, Campina Grande 58429-500, Paraíba, Brazil; 4 Academic Unit of Physics, Federal University of Campina Grande, Campina Grande 58429-900, Paraíba, Brazil; viafonso@df.ufcg.edu.br; 5 Academic Unity of Mechanical Engineering, Federal University of Campina Grande, Campina Grande 58429-500, Paraíba, Brazil; antonio.gilson@ufcg.edu.br

**Keywords:** *Psilocybe*, psilocybin, psilocin, indole alkaloids, tryptamines

## Abstract

The *Psilocybe* genus is known for producing tryptamine alkaloids, specifically the compounds psilocybin and psilocin, which have shown antidepressant and anxiolytic potential. The presence of these alkaloids makes *Psilocybe* mushrooms promising sources of molecules with potential applications in the treatment of mental disorders. To explore this, a bibliographic study was conducted with the aim of synthesizing published data regarding the biological properties and chemical composition of *Psilocybe* mushrooms. Searches were performed on indexing platforms, and the articles found were processed using StArt software. These articles were then classified by score and selected based on inclusion and exclusion criteria. This survey yielded a total of 74 articles, and among them, 66 works showed the presence of psilocybin and/or psilocin alkaloids, indicating the psychoactivity of the mushrooms, and 4 works demonstrated the antimicrobial and antioxidant activities of the extract from certain species of the genus. Additionally, 37 chemical compounds were identified across the genus, 23 of which are alkaloids. Data regarding the temporal and chemical stability of these compounds were also observed, which could help optimize the handling of materials that contain indole alkaloids. Therefore, it is evident that species of this genus remain underexplored in terms of chemical diversity; only compounds classified as alkaloids, terpenoids and phenolic compounds were found, and, in total, only 36 compounds in a study range time of 67 years. Furthermore, most studies focused primarily on evaluating the tryptamine alkaloids responsible for the psychoactivity of the mushrooms, without any study focusing on demonstrating the biological activity of isolated compounds against any pathological factor, except for studies relating the whole extract to larvicidal, antimicrobial and antioxidant potential. So, this review provides a general overview of the molecules isolated from the genus and their biological activities and also suggests that researchers working with these mushroom species could focus their efforts on isolating new compounds and evaluating other types of biological activities that can improve the knowledge of mushrooms’ alternative applications.

## 1. Introduction

**Figure 1:**
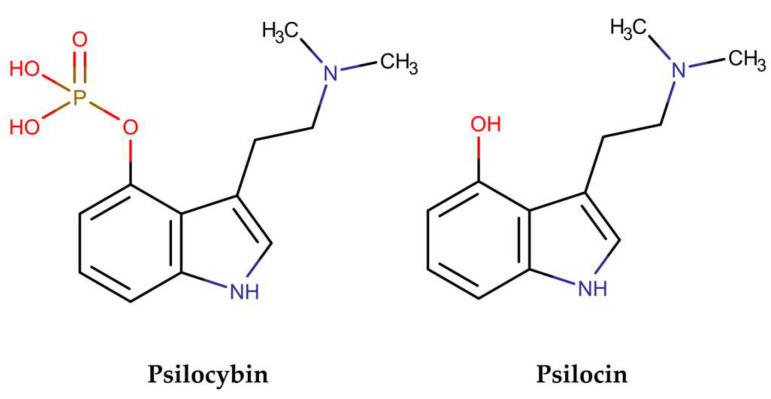
Psilocybin and psilocin found in *Psilocybe* mushrooms.

Mushrooms belonging to the *Psilocybe* genus became popular in the decade of 1950s due to their psychoactive and hallucinogenic properties. Along with their use as a recreational drug and because of the presence of substances with a high structural similarity to serotonin, a neuroreceptor related to mood regulation, the possibility of using them to treat mental illnesses, such as depression, emerged. During this same period, the active compounds responsible for the hallucinogenic effects of the mushroom—psilocybin and psilocin (Figure 1)—were isolated, characterized and synthesized by Hoffman and collaborators [1].

Analysis of the chemical composition of these mushrooms shows the prevalence of these two indole alkaloids as major compounds in most *Psilocybe* species, with psilocybin and its metabolite, psilocin, being responsible for the hallucinogenic effects caused by the consumption of these mushrooms [2]. Studies have demonstrated the ability of these molecules to be used in the treatment of depression and anxiety, as well as an adjunct in the treatment of tobacco and alcohol addiction [3,4,5]. Furthermore, the activity of these compounds is related to their ability to interact with serotonin neuroreceptors, primarily those of the 5-HT1 and 5-HT2 families, due to the structural similarities of these compounds.

However, there are strict regulations regarding studies involving both psilocybin and psilocin, as well as mushroom species of the *Psilocybe* genus. Both substances are classified as controlled substances under the Drug Enforcement Administration (DEA) Schedule I (drugs with no currently accepted medical use and a high potential for abuse), and are also listed in the Canadian government’s inventory of controlled illegal drugs [6,7].

Due to these legal restrictions, studies on the chemical composition of mushrooms from this genus have become difficult, and the primary chemical evaluation conducted in studies identifying the secondary metabolites of these species focuses on determining the levels of indole alkaloids, psilocybin and psilocin, essentially covering the extraction, isolation, quantification and characterization of these alkaloids. The reports of other secondary metabolites are related to some terpenoids found in *P. cubensis* and *P. samuiensis*, lacking the isolation of compounds belonging to other secondary metabolite classes like flavonoids, steroids and phenolic compounds [8,9,10].

There are no reports in the literature on the use of *Psilocybe* mushrooms for the treatment of diseases unrelated to their psychoactivity. Furthermore, the traditional use of these mushrooms, when considering the historical context, is intrinsically linked to the ancient ritualistic and religious practices of indigenous societies [11]. The few reports that show some biological activity that are not correlated with the psychoactivity of these mushrooms describe the antimicrobial, antioxidant and anti-inflammatory activities, but only of two *Psilocybe* species, *P. cubensis* and *P. natalensis* [12,13,14]; other studies to show if these mushrooms are capable of treating cancer diseases or other types of biological activities are lacking.

Given the potential application of *Psilocybe* mushrooms as a source for developing new antidepressant drugs, the number of studies on these mushrooms has increased. It is essential to investigate their chemical, biological, and toxicological properties to identify additional beneficial health effects and to understand the potential adverse effects that may arise from their consumption.

**Figure 2:**
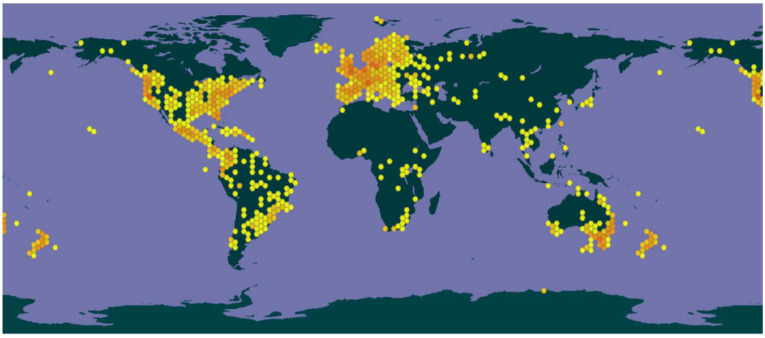
Distribution of Species of the Genus *Psilocybe* on the Globe (GBIF) [16].

The genus *Psilocybe* is the second-largest genus in the Hymenogastraceae family, with 311 mushroom species already catalogued [15]. This genus is distributed across diverse regions of the globe, ranging from the warmest to the coldest areas, with the highest density of occurrences found on the European continent, followed by North America (Figure 2) [16].

**Figure 3:**
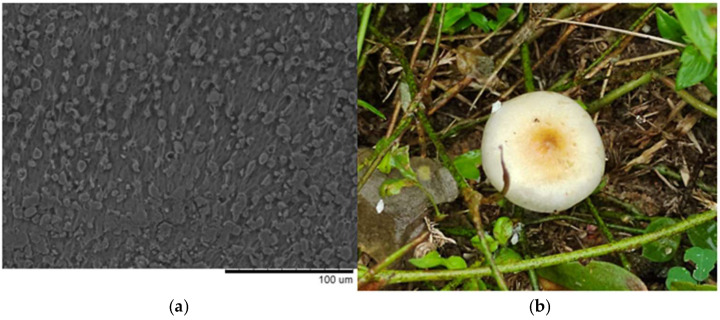
(**a**) Spores of mushrooms of the genus *Psilocybe*; (**b**) *Psilocybe cubensis* mushroom.

Some notable physical characteristics of this genus include the color of the cap, which ranges from white to brown, and the spores, which in some cases may vary from brown to purple (Figure 3).

Despite the growing number of studies on mushrooms of the *Psilocybe* genus, no comprehensive reviews have yet summarized the available literature on their chemical diversity and biological properties. Therefore, the objective of this work is to provide an overview of the chemical composition of *Psilocybe* mushrooms, as well as the biological properties associated with molecules or extracts derived from them.

## 2. Results and Discussion

### 2.1. Secondary Metabolites Found in Mushrooms of the Psilocybe Genus

A bibliographic survey was carried out in which 74 works published between 1958 and 2025 were selected and included in this review. The search terms focused on the identification and/or quantification of chemical substances and biological activities for species of the *Psilocybe* genus. As shown in Table 1, a total of 32 species were studied, with the most prominent being *P. cubensis*, *P. cyanescens*, *P. semilanceata* and *P. bohemica*. These species are cited most frequently in the literature for this genus and are notable for their high levels of psilocybin and psilocin production, as reported in Table 1. Additionally, the vast majority of studies consider the entire mushroom, without distinguishing between its parts for extraction purposes. An exception is found in studies that use the mushroom’s mycelium to extract active compounds, where the product is derived from a growth stage prior to the development of the fully mature fruiting body.

In this bibliographic survey, the major alkaloids reported in the literature were identified as psilocybin and psilocin, with concentration ranges of 0.0008–2.02% and 0.01–1.27% in the mushroom biomass, respectively. Among the species producing these alkaloids in larger quantities, the most notable are *P. cubensis*, *P. baeocystis*, *P. semilanceata* and *P. bohemica*. For psilocybin, the highest levels reported are in *P. cubensis* [10] and *P. semilanceata* [11], both with a maximum content of 2.02%. For psilocin, *P. bohemica* stands out, with a maximum reported content of 1.27% [12], underscoring its potential as an excellent source of these active compounds.

Regarding other alkaloids identified in these species, *baeocystin* appears as the one with the highest concentrations, with levels ranging from 0.001 to 0.11%, while other alkaloids do not exceed 0.05% of the mushroom’s dry mass. Furthermore, after evaluating the literature, no clear relationship between the location of mushroom collection and the concentration of active compounds was found, as collections were conducted in non-standardized locations, and the studies examined species collected from diverse regions.

**Figure 4:**
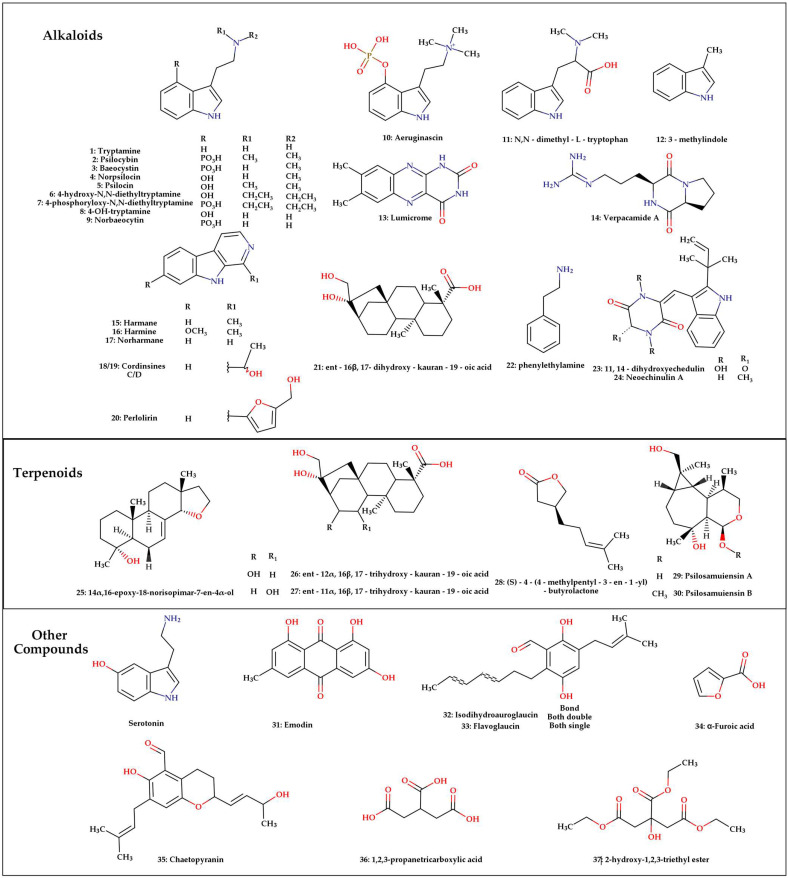
Structure of compounds identified in mushrooms of the *Psilocybe genus*.

In total, 37 substances were identified (Figure 4), with alkaloids being the most studied class of metabolites, accounting for 23 compounds in this review, while the remaining six belong to the terpene class. The summarized data reveal that the majority of studies focused solely on the quantification or identification of psilocybin and psilocin (Table 1). However, 22 studies also characterized additional compounds present in these extracts, such as norpsilocin [17], baeocystin, norbaeocystin and aeruginascin [18]. These are indole alkaloids analogous to the two most common compounds, but, unlike psilocybin and psilocin, they do not exhibit proven hallucinogenic properties directly, but their metabolites can have some capacity to interact with the serotonergic receptors. This highlights the need for exclusive and specific tests on these metabolites to investigate their activity on neuroreceptors related to psilocin, as such studies could potentially reveal alternative therapeutic options for diseases currently associated with psilocybin and psilocin.

Other alkaloids were also identified [19] in mushrooms of the studied genus, including phenylethylamine in *P. semilanceata*. This compound is associated with adverse effects such as tachycardia, nausea and vomiting. The study in question also highlights the low incidence of these adverse effects when isolated psilocybin is consumed, compared to the significantly higher occurrence when the whole mushroom is consumed.

Another notable group of compounds identified are the β-carboline alkaloids, including harmane, harmine, norharmane, harmol, perlolirine and cordysinins C and D (Figure 4), reported in *P. mexicana*. These alkaloids are associated with other tryptamines, such as N, N-dimethyltryptamine, found in the Ayahuasca drink, and are mostly found in plant species like *Banisteriopsis caapi* and *Peganum harmala*; when present in significant amounts, these compounds are responsible for inhibiting the monoamine oxidase enzymes found in the gastrointestinal tract, preventing the oxidation of the tryptamines before the absorption by the organism. This class of compounds is known for inhibiting monoamine oxidase A and B enzymes and may play a role in modulating the effects of tryptamines present in the mushroom. However, these compounds have only been reported once in the literature, with their concentrations in the mushroom being in the microgram-per-gram range of fungal material. This underscores the need for further investigation into this class of compounds to confirm their involvement in the psychedelic activity of these fungi [20].

The other alkaloids identified include N,N-Dimethyl-L-tryptophan, 4-OH-tryptamine, neoechinulin A, verpacamide A and lumichrome [21], which have been reported in the literature to exhibit anticancer activity by inhibiting the growth of human lung cancer cells from the H292 and A549 lines and inducing apoptosis in the cancer cells in question; they are also related with plant growth because of the presence of the compounds in the soil-rhizosphere system [22,23]. Additionally, another alkaloid, 11,12-dihydroxyneoechinulin E, was also identified, but none of these alkaloids have a concrete relationship with the psychoactivity of the mushrooms [8].

Some studies identified the presence of seven terpenoids in mushrooms from the *Psilocybe* genus, including four diterpenes: ent-16β,17-dihydroxy-kauran-19-oic acid [10], which has been reported in the literature to exhibit anti-HIV activity [24]; 14α,16-epoxy-18-norisopimar-7-en-4α-ol, a potential immunosuppressive compound [25]; ent-12α,16β,17-trihydroxy-kauran-19-oic acid [10]; and ent-11α,16β,17-trihydroxy-kauran-19-oic acid [10], associated with anticancer activity against leukemia K562 cell line [10]. Emodin, identified in the metabolic liquid of *P. merdaria* culture [8], has been reported to have antiseptic and anticancer properties [26]. However, it is important to note that these biological activities were not evaluated from compounds isolated direct from *Psilocybe* mushrooms, but rather from molecules obtained from other sources. Additionally, two sesquiterpenes, psilosamuiensine A and B, have been extracted from the metabolic liquid of *P. samuiensis* culture [27], as well as a monoterpene, (S)-4-(4-methylpent-3-en1-yl)-butyrolactone, identified in *P. merdaria* (Figure 4). To date, no biological activity has been reported for any these three compounds [8].

Other compounds found in the *Psilocybe* genus mushrooms were the phenolic compounds isodihydroauroglaucin, which the literature reports as having antimicrobial activity against *Streptococcus pneumoniae* [28], flavoglaucin, chaetopyranin, α-furoic acid, 1,2,3-propanetricarboxylic acid and 2-hydroxy-1,2,3-triethyl ester, but does not have clear biological activities related to them. A fact that needs to be highlighted is that these compounds, with the exception of some indole alkaloids like psilocybin and psilocin, were found in plant extracts and other fungus samples, like flavoglaucin and tetrahydroauroglaucin, an analog of **32**, that are isolated from *Eurotium herbariorum*, and that are related to its radical scavenging activity [29].

As seen, the main class of compounds found in mushrooms belonging to the genus *Psilocybe* are indole alkaloids, which originate from the metabolic process of the amino acid L-tryptophan. It is interesting to note that the biosynthesis process of these compounds is extremely specific in these mushrooms, being found in some species of seven other genera, two of them from the same family as *Psilocybe*, and begins with the decarboxylation of L-tryptophan by the enzyme tryptophan decarboxylase (PsiD). After this, the hydroxylation of the molecule occurs at position 4, catalyzed by the monooxygenase P450 (PsiH). Then, the newly added hydroxyl group is phosphorylated by a kinase enzyme (PsilK). Finally, the process of N-methylation of the nitrogen of the 2-aminoethyl group occurs by the enzyme N-methyltransferase-SAM (PsilM). This process produces tryptamines with different degrees of substitution on the nitrogen atom of the 2-aminoethyl group, as well as molecules that may or may not be phosphorylated on the oxygen atom of carbon 4 of the indole ring [30].

**Table 1:** Fungal secondary metabolites found for the genus *Psilocybe*.

Species	Substances	Mushroom Parts (Type of Extract)	Concentration (%) ^1^	Refs.
*P. arcana*	**2** and **5**	Whole mushroom (methanolic)	**2** = 0.01–1.15 and **5** = 0.03–0.85	[31]
*P. argentipes*	**2** and **5**	Whole mushroom (methanolic)	**2** = 0.125–0.38 and **5** = 0.069	[32,33]
*P. azurescens*	**2**, **5**, **9**, **3**, **4**, **10**, **15**, **16**, **17**, **18**, **19**, **11**, **13** and **14**	Whole mushroom (methanolic)	Not reported	[21]
*P. baeocystis*	**2** and **5**	Whole mushroom (hydro-methanolic)	Not reported	[34,35]
	**2**, **5**, **3** and **9**	Whole mushroom (methanolic)	**2** = 0.15–0.85**, 5** = 0.048–0.3 and **3** = 0.01–0.1	[36,37,38,39,40,41]
*P. bohemica*	**2** and **3**	Cap (methanolic)	**2** = 0.31–1.12 and **3** = 0.02–0.03	[42]
	**2** and **3**	Stipe (methanolic)	**2** = 0.14–0.5 and **3** = 0.01–0.02	[42]
	**2**, **5** and **3**	Whole mushroom (methanolic)	**2** = 0.10–1.34, **5** = 0.01–1.27 and **3** = 0.03	[31,42,43,44,45,46,47,48]
*P. bolivarii*	**2** and **5**	Whole mushroom (methanolic)	Not reported	[49]
*P. bonetii*	**2** and **5**	Whole mushroom (methanolic)	Not reported	[49]
*P. caerulipes*	**2** and **5**	Whole mushroom (methanolic)	Not reported	[39]
*P. coprophila*	**2** and **5**	Whole mushroom (methanolic)	Not reported	[36]
	**11** and **1**	Mushroom mycelium (0.1 M HCl)	Not reported	[50]
*P. cubensis*	**2**, **5**, **3**, **9**, **4**, **10**, **6**, **7**, **15**, **16**, **17**, **18**, **19**, **20**, **11**, **13** and **14**	Whole mushroom (methanolic)	**2** = 0.01–1.35, **5** = 0.01–0.78, **3** = 0.05–0.11, **9** = 0.01–0.02, **10** = 0.01–0.05, **15** = 0.002, **7** = 0.02–0.8% and **6** = 0.2–3.3%	[4,18,20,21,36,41,51,52,53,54,55,56,57,58,59]
	**21**, **26** and **27**.	Metabolic liquid (ethyl acetate)	**21** = 21.1%; **26** = 8.3%; **27** = 2.8%	[9,10]
	**2** and **5**	Cap (methanolic)	**2** = 0.102–0.76 and **5** = 0.0415–0.836	[60]
	**2** and **5**	Whole mushroom (ethanolic)	**2** = 1% and **5** = 0.16%	[45]
	**2**, **5** and **4**	Whole mushroom (hydro-methanolic)	**2** = 0.13, **5** = 0.03 and **4** = 0.0015	[17,61]
	**2** and **5**	Whole mushroom (acid trichloroacetic acid 10%)	**2** <0.005% and **5** <0.005	[62]
*P. cubensis*	**2** and **5**	Whole mushroom (chloroform)	Not reported	[63]
*P. cyanescens*	**2**, **5**, **9**, **3**, **4**, **10**, **15**, **16**, **17**, **18**, **19**, **11**, **13** and **14**	Whole mushroom (methanolic)	**2** = 0.1–1.84, **5** = 0.06–0.76 and **3** = 0.004–0.04	[21,31,35,36,41,46,47,64,65]
*P. fimetaria*	**2**	Whole mushroom (methanolic)	Not reported	[66]
*P. inquilina*	**2** and **5**	Whole mushroom (methanolic)	Not reported	[36]
*P. McKennaii*	Not reported	Whole mushroom (methanolic)	Not reported	[67]
*P. medullosa*	**2** and **5**	Whole mushroom (methanolic)	Not reported	[68]
*P. merdaria*	**23**, **28**, **24**, **32**, **33**, **35**, **25**, **31**, **34**, **36** and **37**	Metabolic liquid (ethyl acetate)	Not reported	[8]
*P. mexicana*	**2**, **5**, **9**, **3**, **4**, **10**, **15**, **16**, **17**, **18**, **19**, **11**, **13** and **14**	Whole mushroom (methanolic)	**2** = 0.25	[1,20,21,69]
*P. montana*	**2** and **5**	Whole mushroom (methanolic)	Not reported	[36,70]
*P. pelliculosa*	**2**, **5** and **3**	Whole mushroom (methanolic)	**3** = 0.007–0.04 and **2** = 0.12–0.71	[36,41]
*P. pseudobullacea*	**2** and **5**	Whole mushroom (methanolic)	Not reported	[70]
*P. samauiensis*	**2**, **5** and **3**	Whole mushroom (methanolic)	0.23–0.90 for all substances	[71]
	**29** and **30**	Metabolic fluid (ethyl acetate)	Not reported	[27]
*P. semilanceata*	**2**, **5**, **3**, **9**, **10** and **22**	Whole mushroom (methanolic)	**2** = 0.02–1.70, **22** = 0.00001–0.000145, **5** = 0.01–0.90, **3** = 0.02–1.10, **9** = 0.077 and **10** = 0.022	[19,31,36,46,47,48,51,52,54,66,72,73,74,75,76,77,78,79]
	**2**	Whole mushroom (methanol with ammonium nitrate)	**2** = 0.55–2.37	[80,81]
	**2**, **5** and **3**	Cap (methanolic)	**2** = 0.33–1.35, **3** = 0.12 and **5** = 0.04–0.68	[48,78]
	**2** and **3**	Whole mushroom (hydroalcoholic acidified)	**2** = 1.09–1.19 and **3** = 0.29–0.41	[82]
	**2**, **5** and **3**	Whole mushroom (methanol: chloroform)	Not reported	[83]
*P. serbica*	**2**, **5**, **9**, **3**, **4**, **10**, **15**, **16**, **17**, **18**, **19**, **11**, **13** and **14**	Whole mushroom (methanolic)	Not reported	[21]
*P. silvatica*	**2**, **5** and **3**	Whole mushroom (methanolic)	**3** = 0.004–0.02	[41,68]
*P. strictipes*	**2**	Whole mushroom (methanolic)	Not reported	[39]
*P. stuntzii*	**2** and **5**	Whole mushroom (methanolic)	**2** = 0.04–0.36, **5** = 0.006–0.012 and **3** = 0.002–0.02	[36,41]
	**2** and **5**	Whole mushroom (hydro -methanol)	Not reported	[84]
*P. subaeruginosa*	**2** and **5**	Whole mushroom (methanolic)	**2** = 0.45–1.41 and **5** = 0.011–0.038	[85,86,87]
*P. subcubensis*	**2** and **5**	Whole mushroom (methanolic)	**2** = 0.32 and **5** =0.06	[32]
	**2** and **5**	Whole mushroom (chloroform)	**2** = 0.80–0.86 and **5** = 0.02–0.03	[88]
*P. tampanensis*	**2** and **5**	Whole mushroom (methanolic)	**2** = 0.01–0.19 and **5** = 0.01–0.03	[54]
*P. wrightii*	**2** and **5**	Whole mushroom (methanolic)	Not reported	[89]
*P. zapotecorum*	**2**, **5**, **3**, **9**, **10**, **8**, **4**, **10** and **1**	Whole mushroom (methanolic)	**2** = 1.06–3.04, **5** = 0.03–0.65, **3** = 0.0024–0.321, **9** = 0.036–0.271, **4** = 0.024–0.142, **10** = >0.01	[90]

^1^: Mass percentage.

Mushrooms belonging to this genus have also been studied for their potential to catalyze the 4-hydroxylation and methylation of synthetic tryptamines through the fermentation of these molecules by the mushroom mycelium. Additionally, the use of this strategy—fermenting synthetic tryptamines as a starting point to obtain indole alkaloids structurally similar to psilocybin and psilocin—has been observed. The study in question aimed to demonstrate the 4-hydroxylation and 4-phosphorylation capabilities of this mushroom on the indole nucleus, showing that concentrations exceeding 3% of the compounds in dry weight of the mushroom powder were obtained after the process.

It is worth noting that the work did not perform the process in the liquid phase but rather in the solid phase, where the metabolites were extracted from the fruiting bodies of the mushroom [52]. Furthermore, the methylation capacity of *P. coprophila* mycelium was also demonstrated, converting tryptamine to 3-methylindole [50]. This suggests that supplementing mushroom cultures from the *Psilocybe* genus with indole alkaloid intermediates could be a viable approach for enhancing the yields of desired active compounds. Additionally, a liquid-phase fermentation route holds potential for obtaining the indole tryptamine alkaloids psilocybin and psilocin, as well as other structurally similar compounds.

#### Techniques for Extracting Indole Alkaloids from the *Psilocybe* Genus Mushrooms

The main techniques used in the extraction of active ingredients include agitation, maceration and ultrasonic baths. Agitation was cited in twenty-nine of the studies evaluated, with the types of agitators used being shakers, magnetic stirrers and vortex mixers. These methods resulted in psilocybin concentrations greater than 2% for species such as *P. cyanescens*. However, a disadvantage of these techniques is the extraction time required, which in some cases can exceed 12 h [36]. Maceration was cited twenty-eight times, yielding 1.70% psilocybin in the dry mass of the extract for *P. semilanceata*. As with agitation, the main issue with this method is the extraction time required to achieve the indicated yields [46,60]. Finally, the use of ultrasonic baths is also noteworthy, having been mentioned in eleven studies, with yields exceeding 1.70% for *P. semilanceata* [51]. This technique demonstrates the greatest potential for extracting the active ingredients from the mushroom, as it offers high efficiency through its mechanism of breaking the cell walls of the fungus. Additionally, it requires a relatively short processing time, with extraction periods ranging from 60 to 120 min, yielding relatively high amounts [21,57,79].

The primary extracting solvent used in processes involving mechanical agitation is methanol, which yields some of the highest concentrations of active ingredients. Psilocybin concentrations ranging from 1.8% to 2.04% in the dry mass of *P. semilanceata* have been obtained using methanol [31,36]. While other solvent compositions have been explored in the extraction process, none have achieved yields as high as those obtained with methanol. For example, mixtures of small amounts of water with methanol, or ethanol combined with water and hydrochloric acid, resulted in yields of less than 1%, with some concentrations falling below 0.1%. This is likely due to the degradation of active ingredients caused by the acid or the water present in the system [45,49,61,62]. An in-depth study about extraction methods of psilocybin and psilocin from *Psilocybe* mushrooms can be found in Galdino et al. [91].

### 2.2. Stability of Alkaloids of the Psilocybe Genus

Psilocybin and psilocin are the main compounds of the *Psilocybe* genus, which has sparked interest in understanding their stability under various extraction conditions, degradation pathways, and metabolism. Additionally, since other indole alkaloids are also potential active compounds alongside psilocybin, it is crucial to explore their behavior and how extraction conditions may influence these compounds as well.

The first chemical reaction that can influence psilocybin levels is dephosphorylation, which occurs due to hydrolysis in both acidic and alkaline conditions. This reaction generates psilocin, a prodrug in the extractive medium that is highly susceptible to oxidation. The first and only report of acid hydrolysis under controllable reaction conditions was published in 1985 by John F. Casale. In this study, psilocybin was extracted using a solution of acetic acid and water, which was then heated to 70 °C for 10 min. The resulting material was extracted with ethyl ether and recrystallized using a mixture of chloroform and *n*-heptane, yielding pure psilocin. However, the reaction yield was not reported.

**Figure 5:**
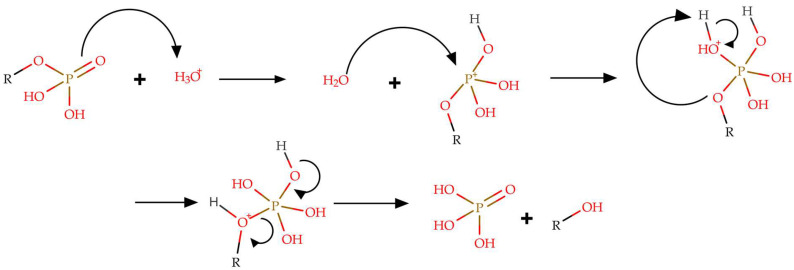
General mechanism proposal for the dephosphorylation reaction.

The reaction mechanism was not addressed in the referenced study [92]. However, when analyzing data related to the dephosphorylation of other alkyl phosphate esters and biological phosphoesters, it was observed that the phosphorus atom is activated through the interaction of the phosphonyl oxygen atom (P=O) with a Lewis acid catalyst (AH or M*^+^*). This activation renders the phosphorus atom susceptible to nucleophilic attack by a water molecule, which subsequently promotes the hydrolysis of such compounds. A generic mechanism for dephosphorylation reactions is illustrated in Figure 5, where psilocybin undergoes a similar reaction process, in a similar way as demonstrated by Hu and collaborators [93]. It is worth mentioning that this reaction can occur in a basic medium; however, the mechanism in such conditions is not well described. In addition, the presence of water in psilocybin extraction systems may lead to hydrolysis, particularly when heat flux is applied, which accelerates the reaction. Other phosphorylated indole alkaloids present in the extract may also undergo dephosphorylation in a manner similar to psilocybin.

**Figure 6:**
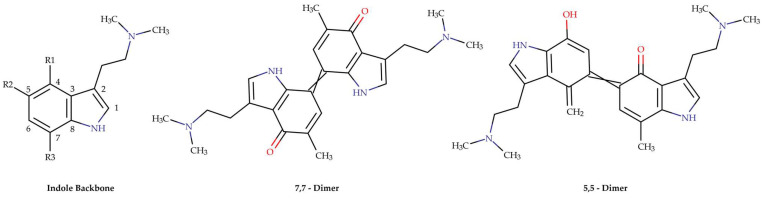
Degradation products of alkaloids of the *Psilocybe* genus.

When a mushroom is collected without undergoing a drying process to eliminate moisture, or if the mushroom is rehydrated, oxidation of psilocybin and psilocin can occur, leading to their conversion into blue-colored polymeric derivatives. This phenomenon is one of the factors that aids in identifying mushrooms containing psilocybin, as physical injury to the mushroom body immediately triggers oxidation, forming these colored products. This reaction is facilitated by the phosphatase PsiP and the laccase PsiL [94]. PsiP catalyzes the dephosphorylation of psilocybin in the first stage, while PsiL oxidizes psilocin in the presence of oxygen and water. During this process, a free radical enters the conjugated system of the molecule via electron delocalization, activating positions 1, 3, 5, 7 and 8 of the indole ring. Due to the steric hindrance and substitution of positions 1, 3 and 8, respectively, only positions 5 and 7 are available for reaction, and the literature indicates that polymeric derivatives form at these positions through the union of two psilocin oxidated radical units [95]. This degradation process is not related to the metabolism of these compounds into therapeutically active forms, but rather results in their complete degradation and the loss of their biological activities. In addition, other degradation products containing up to 13 monomeric units may form, displaying green, orange or blue colors (Figure 6).

The exact function of these colored derivatives remains unknown. However, one prevailing hypothesis suggests that they may be part of the mushroom’s defense mechanism against fungal predators, such as larvae developing alongside the fungus in the substrate, as well as ants and other insects. This hypothesis remains open and requires further studies to establish a definitive relationship between predators and the fungi that produce these colored complexes [96].

In addition, other studies have conducted temporal stability tests on the active ingredients within the fungal matrix, aiming to evaluate the influence of environmental factors on the molecular content over storage time. Gotvaldová et al. [18] carried out experiments to assess various factors, including the temperature to which the mushrooms were exposed, the differences in concentration between dry and fresh fruiting bodies, the impact of freezing on fresh mushrooms, and the effect of storage duration on the concentrations of psilocybin, psilocin and four other structural analogues. The study demonstrated that phosphorylated tryptamines remain stable up to approximately 100 °C, beyond which their concentrations decline sharply. At this temperature, an increase in psilocin concentration was observed, indicating thermal dephosphorylation of psilocybin.

Another aspect evaluated in [18] was the difference in alkaloid content between fresh and dried mushrooms, with no significant changes observed in the indole alkaloid content after drying. The storage of fresh frozen mushrooms at temperatures of −20 °C and −80 °C was found to be highly detrimental to alkaloid content, with psilocybin concentrations decreasing by more than 80% at both temperatures. Conversely, fresh mushrooms stored at 20 °C in a dark environment retained psilocybin concentrations above 1% of the mushroom’s weight after three months. The study suggested that this reduction in concentration was not due to exposure to light or temperature but rather to cutting the mushrooms into smaller pieces while still moist, which promoted oxidation at both storage temperatures. For dried mushrooms, the study showed that the first months of storage exhibit the highest rates of alkaloid degradation, with more than 50% of the original concentration lost during this period. Additionally, it was demonstrated that storage in dark conditions is less harmful to these compounds.

### 2.3. Biological Activities Reported for Extracts of *Psilocybe* Genus Mushrooms

As highlighted, the most notable characteristic of mushroom species from the *Psilocybe* genus is their psychoactivity, primarily due to the presence of the alkaloids psilocybin and psilocin, which act as agonists of the 5-HT2A neuroreceptor. Psilocybin and psilocin readily cross the blood–brain barrier and interact with neuroreceptors, giving them significant potential for treating disorders such as depression, anxiety, and migraines. The psychoactivity of these compounds has been demonstrated through various tests, including the head twich test in mice following consumption of *P. semilanceata* extracts. Additionally, open field tests, the elevated plus maze (EPM) test, and the immobility test in mice show that variable doses ranging from 1–100 mg·kg*^−^*^1^ can induce behavioral changes, potentially related to the anxiolytic effects of the extract [4,79,97]. Furthermore, the literature reports biological activities beyond psychoactivity, with four studies identified in this review that documented such effects. These studies highlighted activities such as antimicrobial and antioxidant properties, suggesting that the *Psilocybe* genus may hold pharmaceutical potential for treating other diseases. However, it is important to note that none of these activities were linked to an isolated compound.

Studies on biological activities other than psychoactivity were found for only three species within this genus. The first, *P. cubensis*, was evaluated for its antibacterial potential against *Escherichia coli*, *Pseudomonas aeruginosa*, *Proteus vulgaris*, *Vibrio cholerae*, *Salmonella typhi*, and *Staphylococcus aureus*. Additionally, *P. cubensis* extracts showed larvicidal activity against mosquito larvae (*Culex quinquefasciatus*) (Table 2), indicating that this species may also have potential for treating infections caused by these pathogens. To access the antibacterial activity of this extract, tests were conducted using agar diffusion and microdilution techniques, with a concentration range of 0.02–10 mg·mL*^−^*^1^, and the larvicidal activity was assessed using the standard method established by the World Health Organization, with a concentration range of 50–800 ppm. Metabolic liquid extract from the *P. cubensis* culture was capable of inhibiting the proliferation of these cultures at the evaluated concentrations, where the lowest inhibition zone diameter was 12 ± 0.1 mm, for *Salmonella typhi*, which was lower than the positive control, Ampicillin, which had an inhibition zone diameter of 17 ± 0.4 mm, but with a MIC = 5.00 ± 0.2 mg·mL*^−^*^1^. The lowest MIC showed in this case was 1.25 ± 0.5 mg·mL*^−^*^1^, for *Proteus vulgaris*, but the MIC of a positive control was not reported to determine if this was a high or low value in the case of this study [9].

**Table 2:** Inhibitory concentrations and type of test using species of the genus *psilocybe*.

Species	Type of Extract	Activity	Strains/Radicals/Others Cellular Mediators	Ref.
*P. cubensis*	Ethyl Acetate (metabolic liquid)	Antimicrobial and larvicidal	*Escherichia coli*, *Pseudomonas aeruginosa*, *Proteus vulgaris*, *Vibrio cholera* (SA), *Salmonella typhi*, *Staphylococcus aureus* and *Culex quinquefasciatus*	[9]
*P. cubensis*	Aqueous	Larvicide	*Artemia franciscana*	[13]
*P. cubensis*	Ethanol and aqueous	Antibacterial	*Escherichia coli* and *Bacillus cereus*	[12]
*P. merdaria*	Metabolic liquid (ethyl acetate)	AChE inhibitor	Acetylthiocholine iodide	[8]
*P. natalensis*	Ethanolic and aqueous	Antioxidant, cytotoxic and anti-inflammatory	Radical ABTS, normal Vero cells, NO inhibition, PGE_2_ inhibition and cytokine inhibition	[14]

Another study indicated the antimicrobial activity associated with the *P. cubensis* extract against the strains *Escherichia coli* and *Bacillus cereus* using the microdilution method, with inhibitory concentrations of 8.192 ppm for both microorganisms (Table 2), which were compared to the positive control, tetracycline, whose minimum inhibitory concentration was 1 ppm. These values shows that the extract has a MIC value eight times higher than the positive control, with this extract promoting a weak antimicrobial activity against both microorganisms [12].

The aqueous extract of the fruiting bodies of *P. cubensis* also demonstrated toxicity against *Artemia franciscana* (Table 2). The test was conducted both for the inhibition of hatching and for the mortality of adult individuals (after 15 days of development). For both cases, a concentration range of 5–500 μg·mL^−1^ was evaluated, which involved adding the extract to the microorganisms and performing a count of individuals after 24 and 48 h. It was observed that a 50% inhibition of hatching for this strain was achieved at a concentration of 135 μg/mL, and for the mortality of adult individuals, it was 172 mg·mL^−1^ [13]. The positive control in this case was potassium dichromate, a very toxic compound, which showed an LC50 of 53 μg/mL, showing that the extract has a medium level of toxicity against this organism.

In all the cases lacking specific information on the use of isolated compounds, the strength and the specificity of the activity cannot be correlated with any compound present in this mushroom. But, in the case of *P. cubensis*, the occurrence of the compound lumichrome was reported in Table 1. This compound can bind with the bacteria and promote its photoinactivation, thus raising the hypothesis of its participation in the antibacterial effect of these extracts, but this activity for the isolated compound is not correlated with *Psilocybe* mushrooms [98].

One of the results that stands out the most is the cholinesterase inhibitor activity showed by one of the compounds isolated from *P. merdaria*, 14α,16-epoxy-18-norisopimar-7-en-4α-ol, which was isolated from ethyl acetate extract of the liquid of mycelium culture. This compound was capable of inhibiting the acetylcholinesterase enzyme at a concentration of 50 μg/mL, showing the capacity of the mushroom to combat Alzheimer’s disease [8].

The hydroalcoholic extract of *P. natalensis* exhibited antioxidant activity through the ABTS free radical scavenging assay with an IC_50_ of 50 µg·mL^−1^. Cellular viability was also determined using the colorimetric tetrazolium-based assay (MTT) at concentrations above 100 µg·mL^−1^, indicating a high cellular viability when compared to the active concentration. This extract also demonstrated positive anti-inflammatory activity against RAW 264.7 cells, for which the concentration range of 10–50 μg·mL^−1^ was evaluated. It also inhibited nitrous oxide (NO) production within the same concentration range for both the aqueous and ethanolic extracts of this mushroom (Table 2) [14]. The author shows that the *P. natalensis* ethanolic extract had the most potent antioxidant activity, but due to the complex mixture of substances that compose this material, is not possible to make the correlation between only one compound and the activity. Other works in the literature show the presence of secondary metabolite classes like flavonoids and phenolic compounds, but this classification was made through colorimetric methods and not by compound isolation, and this can be a possible way by which the extract has the ability to capture free radicals [9].

Two studies were also found regarding the acute toxicity of the extracts from *P. semilanceata* and *P. cubensis* mushrooms in mice [4,79]. For *P. semilanceata*, the extract was compared with pure psilocin, obtaining an LD50 = 293.07 ± 1.02 mg/kg for psilocin. The pure substance showed a relatively lesser safety profile than the mushroom extract, which had an LD50 = 324.37 ± 2.04 mg/kg, both showing medium toxicity [79]. Regarding *P. cubensis*, an acute toxicity of 2000 mg/kg was observed for the aqueous extract, a value much higher than that observed for *P. semilanceata*, making it safer for application. Furthermore, the extract of *P. cubensis* showed no association with pathological processes [4].

Therefore, there is a need for further studies on the biological activities of mushrooms from the *Psilocybe* genus, both to confirm those already described in the literature and to identify new ones, as there are few studies linking species of this genus to biological activities. Furthermore, only a limited number of species have been studied, and none of these studies identify the compounds responsible for the observed bioactive effects. This highlights the opportunity to study the chemical composition of these mushrooms in a targeted manner to discover new compounds with proven biological activity that go beyond the indolic alkaloids and their psychoactive effects.

#### Aspects of the Interaction Between Indole Alkaloids from *Psilocybe* Mushrooms and Serotonin Neuroreceptors

One of the most prominent gaps in the literature is the proof of the mechanism of action of the whole mushroom against the 5-OH-tryptamine neuroreceptors to show that these mushrooms in fact are effective in the treatment of mental diseases. Besides that, is necessary to know how the isolated compounds act in the patient’s body, to what neuroreceptors they bind and which is the most effective in this process. An attempt to summarize what is already known about these topics is made in this discussion.

Some studies have shown how the consumption of *Psilocybe cubensis* mushrooms can change the behavior of the individuals; all studies found conducted tests in mice, and showed how these changes are related to anti-depressant and anxiolytic effects of these mushrooms. Studies show that mice in which they applied the mushroom extract showed changes in their immobility behavior and climbing behavior and a reduced exploration in the open field test, showing that, in fact, the mushroom extracts are capable of promoting some type of interaction with neuroreceptors, but do not show any specific data about the extract influence in specific receptors [99,100,101]. Due to the presence of the indole alkaloids, the serotonergic receptors are the most probable, and the studies shown below give details about which receptors are affected by these molecules and the intensity of this interactions.

The literature reports six indolic alkaloids found in mushrooms of the *Psilocybe* genus: psilocybin, psilocin, baeocystin, norbaeocystin, aeruginascin and norpsilocin, which are studied in an attempt to show their relation with the hallucinogenic activity of these mushrooms, but baeocystin and aeruginascin show a high inhibition factor, showing that only psilocin and norpsilocin are capable of interacting with the neuroreceptors (Table 3). Psilocybin, the alkaloid found in the highest concentration in the *Psilocybe* genus, is the precursor substance to psilocin, the psychoactive molecule of the mushroom. Psilocybin undergoes dephosphorylation upon entering the organism through the action of alkaline phosphatase, the acidic environment in the gastric juice, and forms psilocin, which is rapidly absorbed by the body.

Upon entering the individual’s body, psilocin acts as a serotonin reuptake inhibitor, which binds non-selectively to serotonergic receptors, having a greater affinity for the 5-HT1A, 5-HT2A and 5-HT2C receptors. This activity is responsible for opening up the entire range of possible applications for psilocin. Among the possible applications for psilocin are its uses in the treatment of anxiety disorders and depression, opening up the possibility of its use as a way to treat treatment-resistant depression and headaches; it can also be employed in the treatment of addiction to various drugs, such as alcohol, nicotine, opioids and cocaine [102].

Baeocystin, an indolic alkaloid analogous to psilocybin, is not capable of inducing hallucinations like psilocin, through its interaction with the 5-HT2A receptor. This conclusion was reached after evaluating the head-twitch response (HTR) in mice, which is indicative of hallucinations mediated by serotonergic receptors. The compound did not show a significant effect on the individuals used in the study in question [103].

However, norpsilocin, a product of the dephosphorylation of baeocystin, showed high affinity for the 5-HT1A, 5-HT2A, 5-HT2B and 5-HT2C receptors, acting as an agonist, and demonstrated superior activity to psilocin for the first two receptors (Table 3), while for the other receptors, the inhibition constants were similar to each other [104]. Nevertheless, secondary amines tend to be more rapidly degraded by monoamine oxidase enzymes, which could explain the lack of response to the consumption of its precursor, baeocystin, in the head-twitch response tests [103].

**Table 3:** Tryptamine inhibition factors.

Substance	Receivers	Positive Control	Inhibition Factor (^a:^ Mice; ^b:^ Human; nM)	Ref.
Psilocybin	5-HT1A/2A; 5-HT2B/2C	Ketanserin, LSD and serotonin	^1A:^ 197 ^a^; ^2A:^ 2096^a^ ; ^2B:^ 612 ^b^; ^2C:^ 3741 ^b^	[104]
Psilocin	5-HT1A/2A; 5-HT2B/2C		^1A:^ 118 ^a^; ^2A:^ 235 ^a^; ^2B:^ 8 ^b^; ^2C:^34 ^b^	
Baeocystin	5-HT1A/2A		^1A:^ 25 ^a^; ^2A:^ >5000 ^a^	
Norbaeocystin	No data	No data	No data	
Norpsilocin	5-HT1A/2A; 5-HT2B/2C	Ketanserin, LSD and serotonin	^1A:^ 29 ^a^; ^2A:^ 706 ^a^; ^2B:^ 13 ^b^; ^2C:^ 32 ^b^	
Aeruginascin	5-HT1A/2A		^1A:^ >10.000 ^a^; ^2A:^ >5000 ^a^	

The superscripts with uppercase letter are related to the receptor that the compound binds.

As for norbaeocystin, an analogue of psilocybin but with a primary amine, it does not present hallucinogenic activity, primarily due to its inability to cross the blood–brain barrier, preventing it from entering the central nervous system. This is due to its low partition coefficient (LogP = −2.6), indicating that the molecule is highly hydrophilic and reducing its ability to permeate this membrane, and secondly, due to the ease with which it is degraded by monoamine oxidase enzymes, similarly to norpsilocin [103].

Aeruginascine, a quaternary amine similar to psilocybin, is not as well studied as its counterparts. It is a precursor to the substance 4-hydroxy-N,N,N-trimethyltryptamine, its possible active form, which is obtained through the dephosphorylation of aeruginascine, a compound that has not been identified as present in mushrooms of the *Psilocybe* genus. Studies show that the dephosphorylated substance has a good ability to bind to the same serotonergic receptors as psilocin, with lower inhibition constants, though it is not as effective as psilocin. Furthermore, it is also shown that aeruginascine does not bind as strongly as psilocin to the 5-HT_2B_ receptor, which may be related to the development of cardiac complications [105].

## 3. Materials and Methods

**Figure 7:**
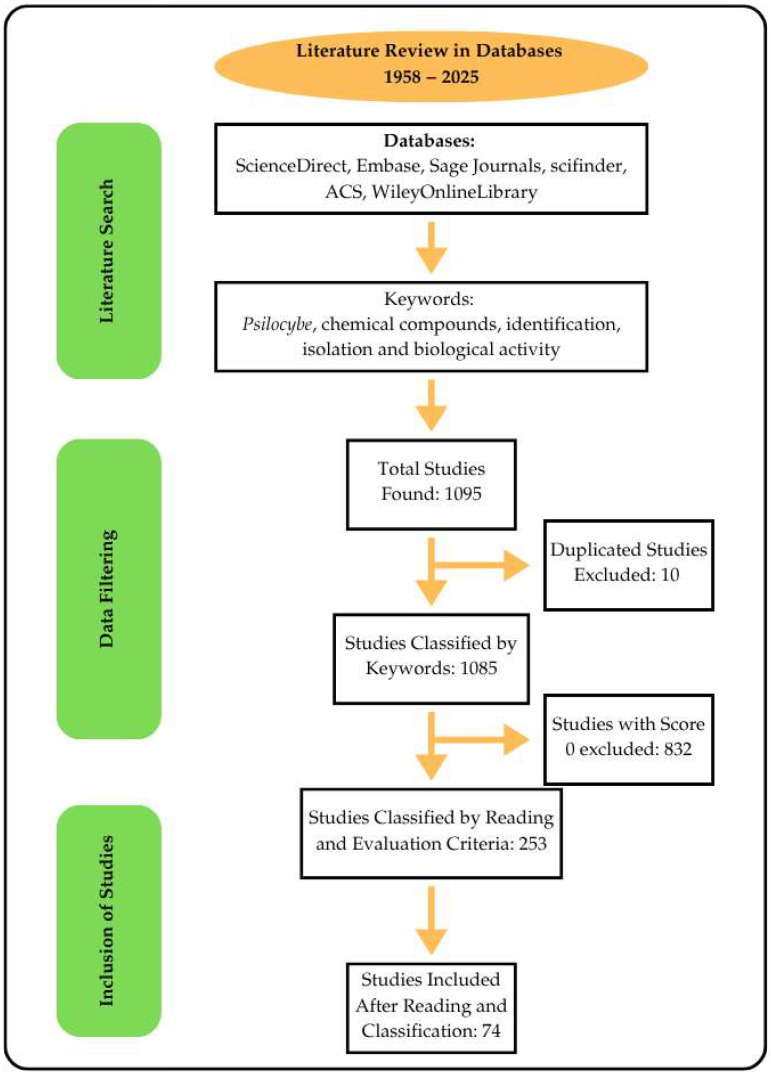
Literature review protocol.

In this work, a literature review was conducted on published works from 1958 to May 2024. For this, a protocol containing the keywords *Psilocybe*, Chemical compounds, Identification*,* Isolation *and* Biological activity was used. Articles in English, Portuguese, Spanish and French were selected, and the bibliographic search was carried out using the platforms ScienceDirect, Embase, Sage Journals, Scifinder, ACS and WileyOnlineLibrary. Initially, 1065 articles were found that matched the search string. With these articles, data processing was performed based on scoring, where points were assigned to the occurrence of the keywords in the title and abstract of the articles. Articles with a score of 0 were excluded from the analysis. Data selection was performed using the StArt software (Figure 7).

For the selection of the works included in this review, the inclusion and exclusion criteria shown in Table 4 were observed.

After the selection, the articles that met the acceptance criteria were read, and the information contained in these works was compiled and treated to extract only the most relevant data to this research.

## 4. Conclusions

This is the first review summarizing the available literature reporting the chemical constituents and biological properties of extracts from mushrooms of the *Psilocybe* genus. Throughout this review, the isolation of only 26 molecules, 20 alkaloids and 6 terpenoids, from mushrooms of the genus *Psilocybe* was identified. The indole alkaloids psilocybin and psilocin are the most studied, followed by norpsilocin, baeocystin, nor-baeocystin and aeruginascin. These mushrooms produce psilocybin concentrations that can exceed 2% of the compound by dry mushroom weight, while psilocin concentrations have been observed to reach up to 1.27%. Furthermore, solid-state fermentation for the production of these mushrooms proves advantageous due to the need for relatively inexpensive inputs, and substrate supplementation with tryptophan derivatives can be used to increase psilocybin and psilocin concentration levels.

This work identifies the existence of gaps in the literature regarding the biological activities and chemical diversity of the *Psilocybe* genus. Due to the complex mixture of substances of which the extract is composed, its use may cause other effects in the patient’s body after consumption, in addition to their psychoactivity and psychological benefits. It may cause interference in the intestinal microorganisms, for example, or the substances present in its composition may interact with other receptors, such as those present in cancer cells or those related to inflammatory processes, but the literature does not show any clear relation between any compound present in these extracts and any other biological receptor related to the patient’s body. On the other hand, it was observed that, in addition to the psychoactive effects, the total extract of mushrooms from this genus also exhibits cytotoxic, anti-inflammatory, larvicidal and antimicrobial properties, but these activities were not yet directly linked to specific compounds; this shows both main gaps existing in the literature related to this mushroom genus: the lack of knowledge about the secondary metabolite composition of this material, and the link between these compounds and the main and secondary biological activities that these mushroom extracts sparsely exhibit currently.

Finally, with this research we hope to assist the comprehension of the current data about the chemical composition of the mushrooms from the *Psilocybe* genus, and show that there is a wide range of molecules inside these types of fungal materials of which there is no knowledge of the structure or biological properties, and thus show that there is room for the development of new applications of this mushroom genus in addition to its application in mental illness treatment.

## Figures and Tables

**Table 4:** Criteria for inclusion and exclusion of articles evaluated in this research.

Inclusion Criteria	Exclusion Criteria
Experimental works	Articles with score 0
Articles presenting the isolation of compounds from species of the *Psilocybe* genus	Articles that are not experimental
Articles presenting biological activities for species of the *Psilocybe* genus	Articles lacking the keywords in the text
	Forensic analysis of human biological samples

## Data Availability

Not Applicable.
